# Supplementary material for: Parental Views on the Acceptability and Feasibility of Measurement Tools Used to Assess Movement Behaviour of Pre-School Children: A Qualitative Study
Source: Int J Environ Res Public Health. 2022 Mar 21;19(6):3733. doi: 10.3390/ijerph19063733 (PMC8949363; doi:10.3390/ijerph19063733)
Supplement: Supplementary file 1 [file ijerph-19-03733-s001.zip › Parental views of measurement tools_Supplementary files.pdf]

Supplementary file S1. *Images and information of the devices*

Actigraph GT3X+ (ActiGraph, Pensacola, USA) is a triaxial accelerometer. The size of the Actigraph GT3X+ is 4.6 cm × 3.3 cm × 1.5 cm and weighs 19g.

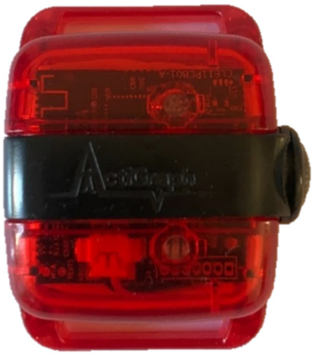

activPAL4 micro (PAL Technologies Ltd., Glasgow, UK) is an uniaxial accelerometer and inclinometer. The size of the activPAL is 2.35cm x 4.3cm x 0.5 cm and weighs 9.5g.

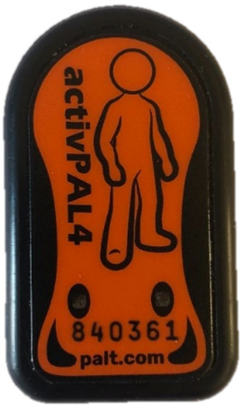

Actical (Philips Respironics Inc., Murrysville PA, USA) is an omnidirectional accelerometer. The size of the Acitcal is 2.8 cm x 2.7cm 27 x 1 cm and weighs 17 g.

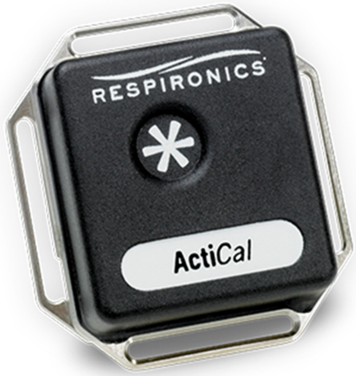

Supplementary file S2. *Instructions for devices (Actigraph GT3X+ and activPAL4 micro examples)*

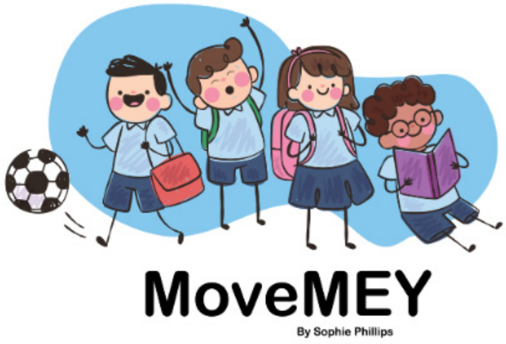

What is it?

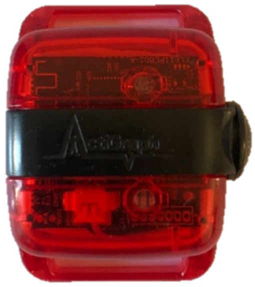

It is called an 'Actigraph' and it measures the physical movement of your child, including when they are physically active, sitting down or lying down.

How does it work?

It measures how much and how fast your child's body moves up and down. It does not need to be switched on or off as it is already set and ready to go. Don't worry if it flashes or not it will be working!

How does my child wear it?

It should be worn around your child's waist for 7 days including a weekend. Try to make sure that the monitor sits on the right side of your child's hip and it is next to their skin and under their clothes.

When does my child wear it?

It is very important for the monitor to be worn 24 hours of the day. The monitor is waterproof but it is best to take off and put it in a safe place if your child has a bath or a shower or goes swimming. Please try to remember to put it back on immediately afterwards, and try to remember to record the time it is taken off and put back on in the table on the next page.

What if I have any questions about the monitor?

If you have any questions, you can contact Sophie Phillips (sophie.m.phillips@durham.ac.uk).

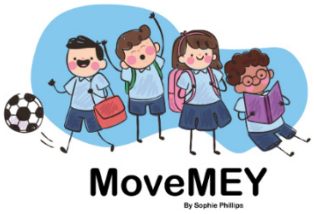

MoveMEY

By Sophie Phillips

What is it?

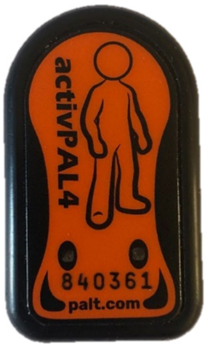

It is called an 'ActivPAL' and it measures the physical movement of your child, including when they are physically active, sitting down or lying down.

How does it work?

It measures how much and how fast your child's body moves up. It does not need to be switched on or off as it is already set and ready to go. The monitor will flash green - this means it's working!

How does my child wear it?

The monitor should be worn on the front of the thigh, roughly 1/3 of the way between the hip and knee with the stick man standing up. The monitor should be kept in the waterproof sleeve, and attached directly onto the skin, and covered with an adhesive patch. It should be worn for 7 days including a weekend.

When does my child wear it?

It is very important for the monitor to be worn 24 hours of the day. The monitor is waterproof so your child can wear it whilst showering and bathing, but it is best to take it off if your child goes swimming. Please try to remember to put it back on immediately afterwards, and try to remember to record the time it is taken off and put back on in the table on the next page.

What happens if I need to change the adhesive patch?

1. Remove the thigh monitor and peel off the adhesive patch.
2. Wipe down the monitor and the area of your thigh with the alcohol prep pad
3. Position the monitor in the same place, ensuring the stick man on the front is standing up (as can be seen in the image on this sheet).
4. Place the new adhesive patch over the monitor and gently press this down to ensure it is secure.

What if I have any questions about the monitor?

If you have any questions, you can contact Sophie Phillips (sophie.m.phillips@durham.ac.uk)

MoveMEY

By Sophie Phillips

Supplementary file S3. *Stages of thematic analysis.*

Stage of analysis	Analysis conducted
Familiarisation	Collecting the data through focus group discussions and reading through the transcripts multiple times over several months.
Initial coding generation	Line by line coding of each transcript, followed by creating a document outlining the codes, notes, and relevant extracts of the data; to show where extracts and notes from the data could be grouped under the coded subheadings.
Identifying key themes	Grouping together relevant codes from within and across transcripts. Relevant codes (and accompanying extracts of data) were grouped when they were on a similar topic to create some main themes. This staged helped to consider how different codes could be combined and grouped together under overarching themes.
Reviewing of the themes	Reviewing original codes and data extracts to ensure the overarching theme adequately captured the coded data, alongside reading through the original transcripts to ensure that the developed themes were reflective of the original data, and that each theme contained the relevant and appropriate extracts of data from the transcripts.
Theme definition and labelling	The final stage included defining and labelling the themes by revisiting all phases of analysis and ensuring that each of the key themes captured the essence of the data.

Supplementary file S4. *Infographic of recommendations*.

See powerpoint
