# Supplementary material for: Parental Views on the Acceptability and Feasibility of Measurement Tools Used to Assess Movement Behaviour of Pre-School Children: A Qualitative Study
Source: Int J Environ Res Public Health. 2022 Mar 21;19(6):3733. doi: 10.3390/ijerph19063733 (PMC8949363; doi:10.3390/ijerph19063733)
Supplement: Supplementary file 1 [file ijerph-19-03733-s001.zip › Supplementary File S4_Infographic of Recommendations.pdf]

10 recommendations for measuring movement behaviours of pre-school children

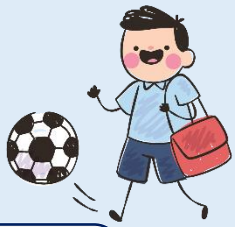

1. Context is important to parents and carers– having space to explain their child's health status (e.g. illness) helps make measurement meaningful.

2. Ensure that measurement captures different routines to be reflective of children's movement e.g. home vs school, weekday vs weekend.

3. Devices worn as watches or placed 'out of sight' were preferred. Devices stuck to the skin were less favourable.

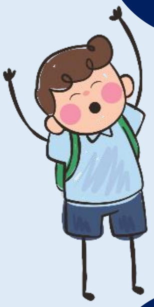

4. Smaller devices preferred for 24 hour movement measurement, but larger devices may be preferred for day time measurement only and smaller devices for night time.

5. Removal of devices may be inevitable so include detailed device wear time logs with plenty of space to report when the device is worn/removed.

6. 'Child-friendly' devices – suitable for playing in mud, slime, sand and water, and no small parts! Demonstrate safety of devices prior to studies.

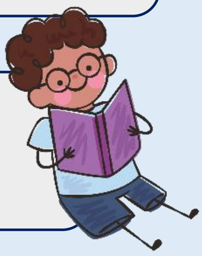

7. Written and visual (video demonstrations) study instructions most helpful.

8. Frame research to young children so that taking part is 'cool' – devices can give 'superpowers' or 'magic'.

9. Modify tools to make them 'childlike' e.g. so children can personalise their device with stickers or provide devices with colourful belts or with children's favourite characters on.

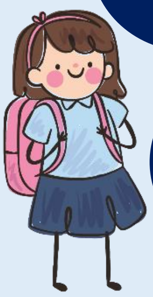

10. Daily reporting easier for parental reported tools – recall can be particularly challenging with young children. Ensure that age appropriate activities are included on the tool.

Source: Phillips, S.M.; Summerbell, C.; Hesketh, K.R.; Saxena, S.; Hillier-Brown, F.C. Parental Views on the Acceptability and Feasibility of Measurement Tools Used to Assess Movement Behaviour of Pre-School Children: A Qualitative Study. *Int. J. Environ. Res. Public Health*, **2022**

NIHR | School for Public Health Research
